# Major areas of interest of artificial intelligence research applied to health care administrative data: a scoping review

**DOI:** 10.3389/fphar.2022.944516

**Published:** 2022-07-18

**Authors:** Olga Bukhtiyarova, Amna Abderrazak, Yohann Chiu, Stephanie Sparano, Marc Simard, Caroline Sirois

**Affiliations:** ^1^ Faculty of Pharmacy, Université Laval, Québec, QC, Canada; ^2^ Quebec National Institute of Public Health, Québec, QC, Canada; ^3^ Faculty of Medicine, Université Laval, Québec, QC, Canada

**Keywords:** artificial intelligence, health care administrative database, pharmacotherapy, scoping review, natural language processing

## Abstract

**Introduction:** The ongoing collection of large medical data has created conditions for application of artificial intelligence (AI) in research. This scoping review aimed to identify major areas of interest of AI applied to health care administrative data.

**Methods:** The search was performed in seven databases: Medline, Embase, CINAHL, Web of science, IEEE, ICM digital library, and Compendex. We included articles published between January 2001 and March 2021, that described research with AI applied to medical diagnostics, pharmacotherapy, and health outcomes data. We screened the full text content and used natural language processing to automatically extract health areas of interest, principal AI methods, and names of medications.

**Results:** Out of 14,864 articles, 343 were included. We determined ten areas of interest, the most common being health diagnostic or treatment outcome prediction (32%); representation of medical data, clinical pathways, and data temporality (i.e., transformation of raw medical data into compact and analysis-friendly format) (22%); and adverse drug effects, drug-drug interactions, and medication cascades (15%). Less attention has been devoted to areas such as health effects of polypharmacy (1%); and reinforcement learning (1%). The most common AI methods were decision trees, cluster analysis, random forests, and support vector machines. Most frequently mentioned medications included insulin, metformin, vitamins, acetaminophen, and heparin.

**Conclusions:** The scoping review revealed the potential of AI application to health-related studies. However, several areas of interest in pharmacoepidemiology are sparsely reported, and the lack of details in studies related to pharmacotherapy suggests that AI could be used more optimally in pharmacoepidemiologic research.

## Introduction

Healthcare sectors generate a huge amount of complex data such as hospital records and examination results, medical insurance claims, and data from medical imaging and monitoring with medical devices (Raghupathi and Raghupathi, 2014). The emergence of digitized data provides a radical improvement of the health system in terms of efficiency and costs (Khan et al., 2019). Health care administrative database has gained particular interest in research, and notably in pharmacoepidemiology. These data are primarily collected by government institutions or other types of organizations and represent a rich source of information (Kone Pefoyo et al., 2009). They are classically generated through a physician’s office registration, a prescription transaction and record keeping at a community pharmacy, an admission to hospital, or a delivery of diagnostic service (Timofte et al., 2018). Health care administrative databases are typically vast, covering very large population samples over a lengthy period. The fact that this type of data is regularly and continuously collected through a consistent way without requiring extra resources is a significant advantage for research and allows for more advanced research questions (Timofte et al., 2018). Furthermore, health care administrative databases have been successfully used for disease surveillance (Blais et al., 2014). As those databases get larger and technology improves, new analysis methods are needed to exploit them correctly.

Artificial intelligence (AI)-based algorithms and tools such as deep learning, machine learning, and reinforcement learning have been successfully used in a variety of health data research and development projects (Bates et al., 2014). AI-based strategies have been designed to transform data into meaningful and actionable insights that help stakeholders to take action or make a clinical decision (Raghupathi and Raghupathi, 2014). For example, AI can be used to predict outcomes, understand pharmacotherapy, or evaluate spatio-temporal models. Compared to classical statistical techniques, novel AI-based strategies can be more accurate, efficient, precise, and impactful (Gubbi et al., 2013; Mehtaa et al., 2019), though this is not always the case, depending on the context (Sessa et al., 2021). The potential of health care administrative databases analyzed with this type of tools remains to be elucidated.

This scoping review was conducted to explore the current state of existing research according to the application of AI to health care administrative data, including those involving medications. The objectives were to identify:

1. The major areas of interest of current studies related to the application of AI to health care administrative data.
2. The principal AI methods applied to research involving health care administrative data.
3. Main clinical and pharmacotherapeutic interests of AI-based research.

## Materials and methods

### Overview

**FIGURE 1:**
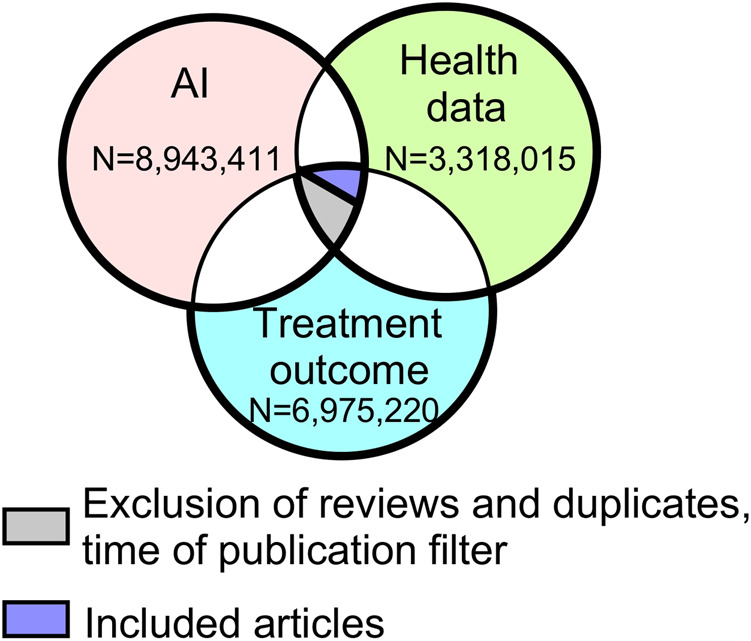
Venn diagram of the initial database search results. Legend: Each circle represents the number of articles related to each topic. There were 14,864 articles that overlapped the three topics. AI, artificial intelligence.

The planning of the search strategy was performed with a research librarian, who was involved at all stages of planning and bibliographic research. The initial search was performed in Medline, Embase, CINAHL, Web of science, IEEE, ICM digital library, and Compendex. We searched for articles published between January 2001 and March 2021, that described original research in AI applied to medical diagnostics, pharmacotherapy, and health outcomes data (Figure 1, additional files). The list of key words and results of the search of each of the seven databases can be found in Supplementary Material.

We performed a three-step procedure for the scoping review: abstract review, full text review, and data extraction. The first two steps were performed with Covidence (Covidence systematic review software, Veritas Health Innovation, Melbourne, Australia. Available at www.covidence.org). Each abstract and full text was reviewed by two researchers (OB, AA, YC, SS, MS). In case of conflict, consensus was reached by a vote of a third researcher or by mutual agreement of at least two team members.

### Abstract review

The reviewed abstracts had to correspond to the following criteria to be included in further steps of the review: 1) be based on original study of real-world data. (Protocols and reviews of previously published studies, theoretical approaches, studies based exclusively on synthetic data, as well as description of architecture of medical information systems (e.g., web-based, cloud-based solutions) were excluded); 2) include application of AI methods. (Studies based on descriptive statistics, pre-defined medical algorithms (rules), and solely regression analysis were excluded); 3) be based on large health databases with an arbitrary threshold of at least 1,000 observations. (Clinical essays and clinical trials were excluded due to the relatively small number of participants and the exclusive use of classical descriptive statistics); 4) be based on data types that can be found in health care administrative databases (clinical diagnoses, prescribed medications, medical procedures, hospital services).

Therefore, we excluded studies that were based **exclusively** on the following types of data: medical imaging (e.g., fMRI, CT, X-ray, PET, cell histology); synthetic data; genomics; time-series data (e.g., continuous recordings of vital signs, ECG, EEG); laboratory tests (e.g., glucose, cholesterol); surveys and questionnaires (e.g., depression scales, surveys on quality of life); social media data; free texts without extraction of data comparable to health care administrative database.

When abstracts did not contain enough information about correspondence to inclusion or exclusion criteria, the article was considered for full text review.

### Full text review

In addition to the criteria described above, at this stage we filtered out items that fell under our exclusion criteria. We excluded documents that were not full-text articles (e.g., abstracts from scientific meetings), duplicate publications, or articles that did not have full description of purpose of the study, methods used, or data types (e.g., use of a “cardiology dataset”).

### Data extraction

We applied two different approaches for data extraction that served for different purposes. In objective 1, we used the *classic approach,* that involved reading the article by at least two reviewers. We determined the principal area of interest for the application of AI methods. Each article was categorized into one or more areas which were defined based on agreement between the reviewers. In objectives 2 (determining the principal AI methods used in the research) and 3 (determining main clinical and pharmacotherapeutic interests of AI-based research), we used the *automated* approach, which was based on Natural Language Processing (NLP), an AI method used for automatic extraction of useful information from free texts. This approach helped to retrieve AI methods applied in the included studies, and health data-related and pharmacotherapy-related terms. The procedure was performed in Python with the use of NLTK (Natural Language Toolkit). It consisted of the following steps: import of titles, abstracts, “Methods” and “Results” sections of the articles, text preprocessing (tokenization, lemmatization, parts-of-speech analysis to retain nouns, substitution for synonyms), creating vocabulary of terms for retrieval, and determining number of articles in which the terms from the vocabularies were used.

### Data analysis

In objective 1, we described the proportion of articles that pertained to the major areas of interest that were identified in the extraction process. In objectives 2 and 3, the frequency of use of the terms was determined based on three different custom-created vocabularies. First, the names of selected statistical and AI methods and their commonly used acronyms were targeted in objective 2. In order to do so, we created a vocabulary of 24 popular AI methods that contained their names (e.g., ‘regression’) and commonly used acronyms (e.g., ‘rnn’ for ‘recurrent neural network’ or ‘svm’ for ‘support vector machine’) based on preliminary screening of the articles. We also investigated evolution of use of AI methods in three-year-long periods from 2001 to 2021.The second custom-created vocabulary was related to the objective 3, and pertained to health data related terms that included clinical diagnoses, terms related to medical and hospital services, and names of socio-demographic variables. Finally, the third custom-created vocabulary was also related to objective 3 and comprised pharmacotherapy-related terms based on Anatomical Therapeutic Chemical (ATC) codes and American Hospital Formulary Service (AHFS) Pharmacologic-Therapeutic Classification System. We manually defined pairs of words that could be considered as synonyms in the context of this study (such as, for example, illness and disease, electronic patient record and electronic health record), and performed replacement of the words that had close meaning with their synonyms. We displayed the frequency of use of health data-related and pharmacotherapy-related terms as WordCloud to provide insights into relative popularity of each term.

## Results

### Overview

**FIGURE 2:**
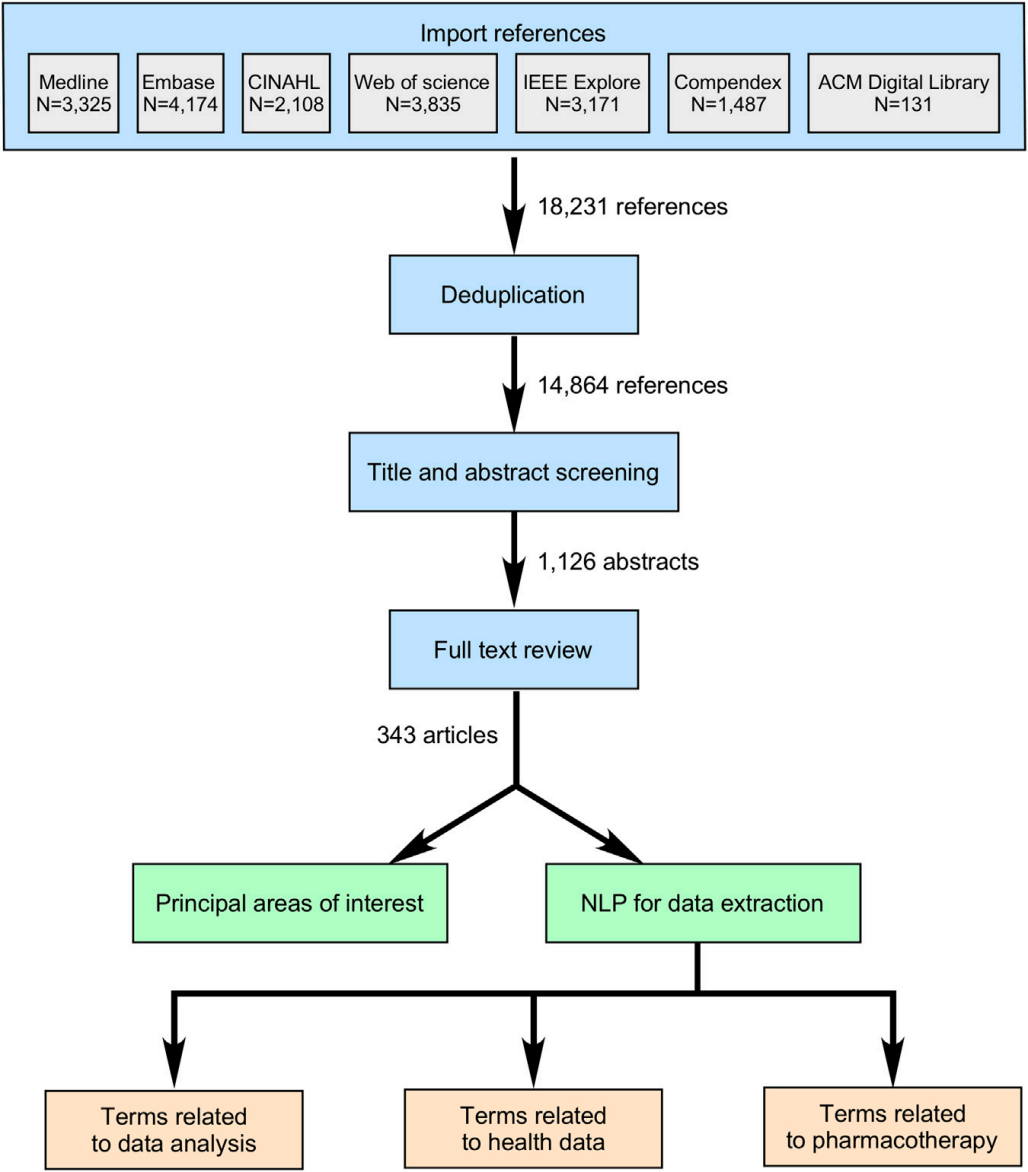
Article selection and data extraction strategy. Legend: The three main steps for article selection included titles and abstract screening, full test reviews, and data extraction. Classic (manual) approach allowed categorizing publications according to the principal areas of applied AI research. Automated method with the use of NLP allowed extraction of terms from three different groups, and their quantification.

Results from our initial database search are presented in Figure 1. After applying the filters for the presence of all three areas of interest, time of publication, and automatic exclusion of reviews and duplicates, the search resulted in 14,864 abstracts (Figure 1, additional files). With further abstract screening based on inclusion and exclusion criteria, the number of articles selected for full text review was narrowed to 1,126, and after their review, 343 articles were included for further analysis (Figure 2). Only 336 articles were included in objectives 2 and 3 due to the exclusion of 7 articles that could not be successfully imported from PDF format.

**FIGURE 3:**
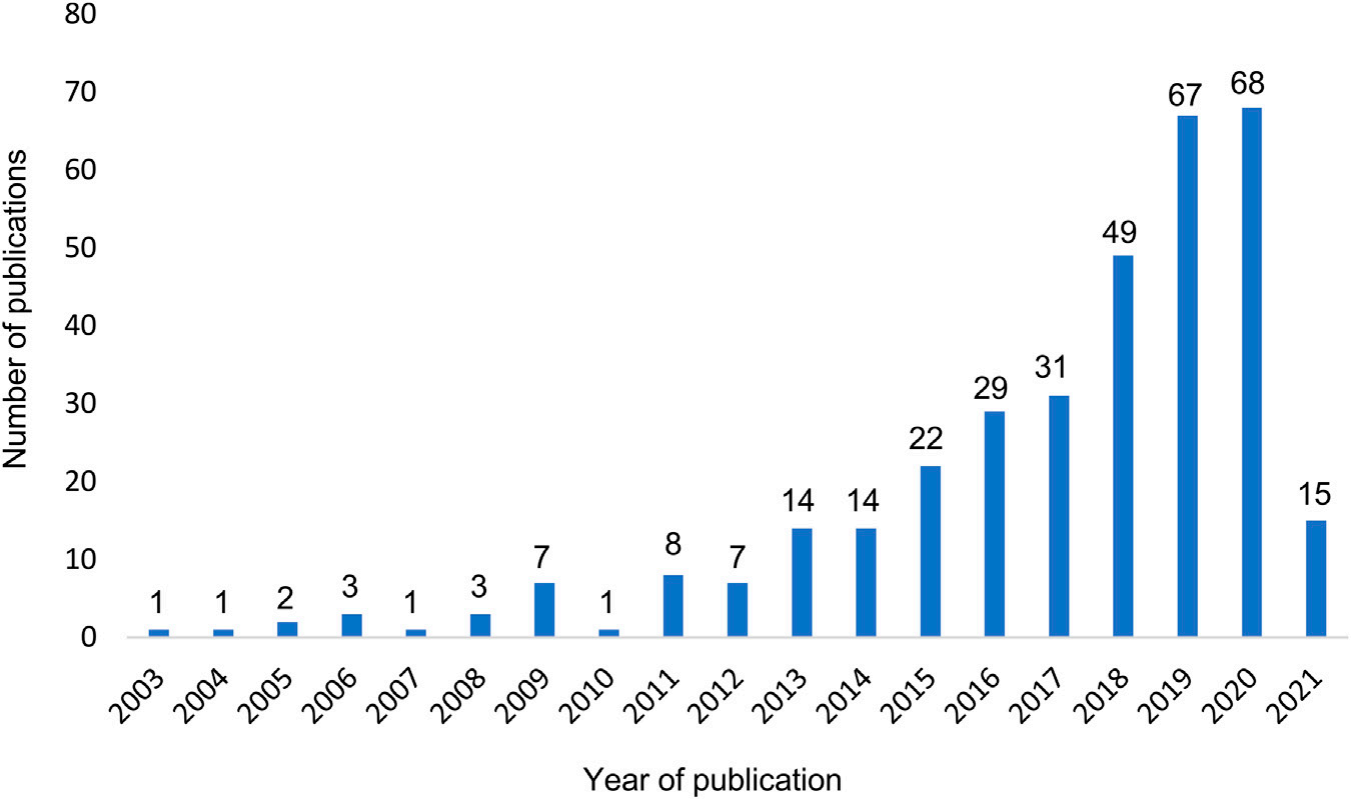
Number of published original articles on AI research applied to health care administrative database per year. Legend: The period 2003–2012 was characterized by a few publications per year and points to an emerging interest in the field. These research topics were rapidly growing in popularity in the following years, most significantly since 2018.

The first full-text articles applying AI to health care administrative databases were published in 2003 (Figure 3). Their frequency increased gradually and reached nearly 70 publications per year in 2020. A proportion of 67% of the articles were published between 2017 and March 2021. More than half of the studies have been published after 2018.

### Principal areas of artificial intelligence application

With the classic review approach, we determined ten principal areas of interest of AI application to health data (Table 1). The most common task for AI methods (N = 111; 32.1%) was to evaluate health state or predict health outcomes, such as predicting hospital length of stay (Azari et al., 2012), readmissions (Yang et al., 2016; Yu and Xie, 2020) or mortality (Melton et al., 2014; Lopez-de-Andres et al., 2016)**.** This category also includes articles that focused on predicting the development of important health problems, such as stroke occurrence within a five-year period (Hung et al., 2017), cancer (Wang H. H. et al., 2019; Wang Y. H. et al., 2019), complications of diabetes (Liu et al., 2020), or adverse outcomes in COVID-19 patients (Shao et al., 2021).

**TABLE 1:** Principal areas of AI application for health care administrative database.

Area of interest	Number of articles	% from total
Health diagnostics or health outcome prediction	110	32.0
Medical data representation, clinical pathways, and temporality	74	21.5
Medication patterns, ADE, DDI	52	15.1
Medical data clustering	47	13.7
Multimorbidity	45	13.1
Combination of medications and treatment patterns	23	6.7
Subpopulations	9	2.6
Polypharmacy	6	1.7
Missing or biased medical data	3	0.9
Reinforcement learning	3	0.9

ADE, adverse drug events; DDI, drug-drug interaction.

The second most common objective of the studies (N = 74; 21.5%) aimed to develop AI for medical data representation including describing clinical pathways and taking into consideration time domain of medical data. These studies used sophisticated mathematical methods applied to longitudinal and heterogenous data (Zhang et al., 2018) to describe sequence of clinical events (Esteban et al., 2015) or inpatient services (Han et al., 2020), or to model disease progression (Powell et al., 2019).

A total of 52 articles (15.1%) focused on medication sequence patterns, adverse drug events (ADE), and drug-drug interactions. Examples of research topics of these studies are specific complications of pharmacotherapy, such as drug interaction effects on myopathy (Chasioti et al., 2019) or role of diabetes medications in the development of renal failure (Davazdahemami and Delen, 2019a; Davazdahemami and Delen, 2019b). This group of articles also includes those that used AI methods to extract information about ADE from such sources as Electronic Health Records (Bagattini et al., 2019) and health care administrative databases (Jin et al., 2008).

A significant share of included studies focused on regrouping medical data. These studies were mostly dedicated to mathematical approaches for: data clustering and evaluation of similarity between patients (13.7%) (Santana-Velásquez et al., 2020), combining analysis of patients with multiple medical diagnoses—multimorbidity (13.1%) (Bueno et al., 2018), or analysis of specific socio-demographic or geographic subpopulations (2.6%).

Only a few articles were using AI to determine combinations of medications in use and treatment patterns (6.7%). The least frequent objectives of the studies were related to polypharmacy (1.7%), to solving problems of missing or biased medical data (0.9%), or to building treatment recommendations based on reinforcement learning (0.9%).

### Principal artificial intelligence methods applied to health care administrative data

The top five most frequently used AI methods were decision trees, cluster analysis, random forests, support vector machines and recurrent neural networks (Table 2). Their popularity started to grow from 2012 to 2014 and has continued to increase until present. Regression and correlation analyses remained widely used in combination with AI methods, mainly for comparison of the research results or for data preparation (e.g., exclusion of highly correlated variables).

**TABLE 2:** Principal AI methods applied to health care administrative database.

Methods	Years						Total (%)
	2003–2005	2006–2008	2009–2011	2012–2014	2015–2017	2018–2020	
Regression	1	2	4	9	35	88	150 (44.6)
Correlation	1	1	5	4	26	52	105 (31.3)
Decision tree	4	0	3	10	28	48	102 (30.4)
Cluster analysis	0	1	3	10	22	58	101 (30.1)
Random forest	0	1	0	3	16	33	59 (17.6)
Support vector machine	0	0	0	4	9	24	41 (12.2)
Recurrent neural network	0	0	0	0	6	32	39 (11.6)
Bootstrap	0	0	1	5	9	18	36 (10.7)
Naïve Bayes	0	0	1	4	6	21	31 (9.2)
Long short-term memory	0	0	0	0	4	24	29 (8.6)
Apriori	3	3	4	5	6	8	28 (8.3)
Boosting	0	0	0	1	2	20	26 (7.7)
k-Nearest neighbors	0	0	1	1	5	15	24 (7.1)
Multi-layer perceptron	0	0	1	0	4	14	21 (6.3)
Principal component analysis	0	0	2	3	3	10	18 (5.4)

Note: Linear discriminant analysis, cox models, hierarchical clustering, autoencoders, hidden Markov models, adaboost, reinforcement learning, generative adversarial networks, and self-organizing maps were mentioned in less than 5% of the articles and thus not included in Table 2.

### Automated extraction of health-related terms from scientific articles

**FIGURE 4:**
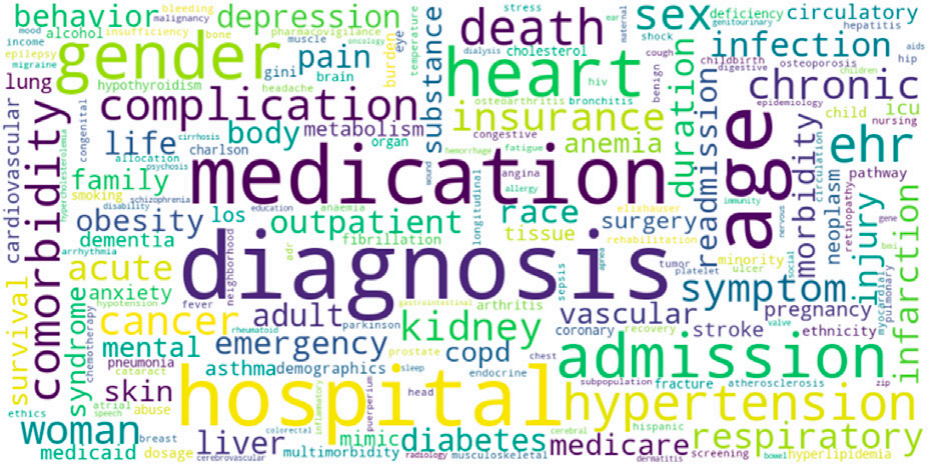
Principal health-related terms mentioned in AI applied studies. Legend: The larger font corresponds to the higher number of research articles where the terms were found.

After performing all the steps of text processing described in the Methods section, we obtained 16,532 unique words that were defined as nouns and a corresponding number of articles in which these words were found. We chose the most frequently used terms related to health data, which resulted in a vocabulary of 190 words (Figure 4).

The word cloud in Figure 4 represents frequency of principal health-related terms extracted from scientific articles. The larger size of the words correlates with the larger number of articles in which the term was used. The terms “diagnosis” (N = 289) and “medication” (N = 215) were among the most frequently used. Most of the data was related to hospitals (N = 220), with particular interest in hospital admissions (N = 121) and emergency rooms (N = 52), while fewer articles focused on outpatients (N = 51). The most common clinical topics of interest were related to heart (N = 146, including hypertension, N = 115), cancer (N = 75) and kidneys (N = 70). Other common topics were respiratory diseases, infections, injuries, pain, and diabetes. Many articles considered not just single diagnosis, but also comorbidities (N = 95). The research was more often focused on chronic diseases (N = 78) than on acute conditions (N = 65). Socio-demographic data was often taken into consideration, with a special emphasis on age (N = 231), gender/sex (N = 135/91, correspondingly), particularly women and race (N = 55).

### Automated extraction of pharmacotherapy-related terms from scientific articles

The reviewed articles were presenting results of interdisciplinary research of AI applied to health. However, they were often lacking in detail on health-related input data and health-related results. In particular, even though many articles used the words “medication” or “drug”, they were commonly missing information of what medications exactly were included in the study.

**FIGURE 5:**
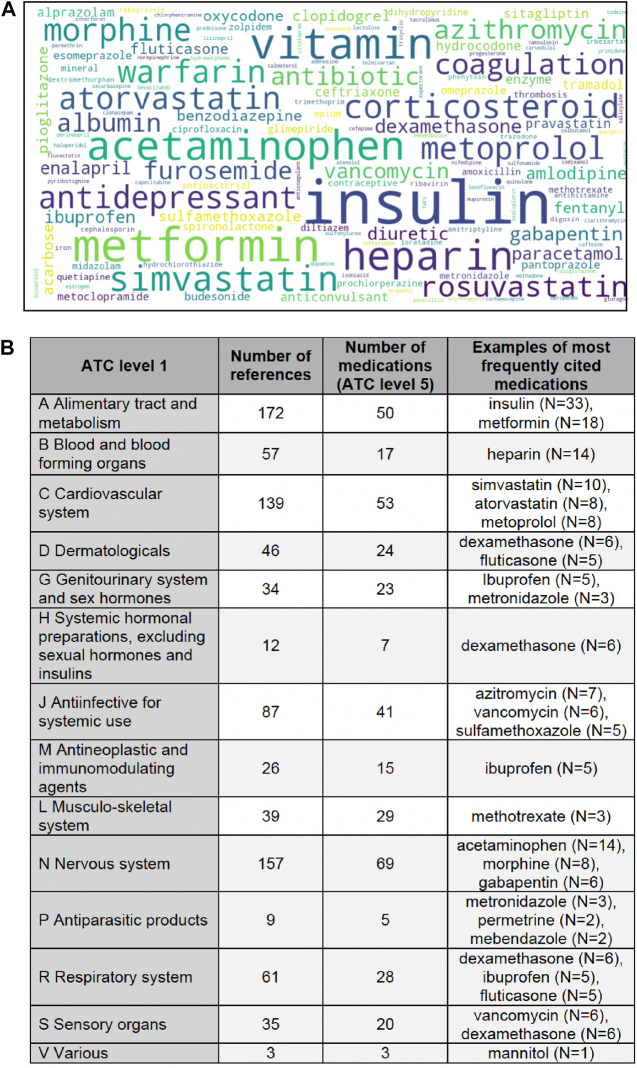
Frequently used pharmacotherapy-related terms. Legend: **(A)** The larger font of the word cloud corresponds to a larger number of articles where the terms were found. **(B)** The table presents information about the number of mentions belonging to ATC level 1 classes, the number of ATC level 5 medications found in the articles, and examples of the most frequently mentioned medications. Some medications may be included in more than one ATC class (for example, dexamethasone appears in classes D, H and R), since the medication indications were not extracted. Some level 1 ATC classes are thus overrepresented (for example, the circumstances in which ibuprofen was studied may not belong to class G (intravaginal use), but rather to class M (anti-inflammatory agent), but ibuprofen has been listed in every ATC class to which it may belong).

Our analytic NLP-based method allowed to directly quantify the use of pharmacotherapy-related terms. We performed automatic extraction of single word terms found in ATC and AHFS classifications, that composed a vocabulary of 1,129 terms. The terms most frequently used are presented in Figure 5. Active substances most frequently mentioned were related to following anatomical and pharmacological groups (ATC level 1): alimentary tract and metabolism (172 mentions of 50 different substances), nervous system (157 mentions of 69 substances) and cardiovascular system (139 mentions of 53 substances). The least represented in the AI-based studies were antiparasitic products (9 mentions of 5 substances) and systemic hormonal preparations excluding insulins (12 mentions of 7 substances). More specifically, the most frequent terms were “insulin” (N = 33), “metformin” (N = 18), “vitamin” (N = 16), “acetaminophen” (N = 14), and “heparin” (N = 14). They were followed by such medication names as simvastatin, warfarin, atorvastatin, morphine, and metoprolol.

## Discussion

The review demonstrated the growing popularity of AI research in health domain, specifically in application to health care administrative data. This phenomenon can be explained by accumulation of large volumes of this type of data, improved access to such databases to AI researchers, further development of AI methods, improving computational capacities, and increased funding of interdisciplinary projects. Application of AI to health care administrative data is heterogenous in terms of areas of interest and methods. We discerned ten areas subdivided into a wide range of sub-areas involving 25 principal statistical and AI methods, 190 terms pertaining to health and 1,129 terms pertaining to pharmacotherapy. We showed that some areas are more popular (e.g., health diagnostics or outcome prediction) while others are still vastly underrepresented (e.g., reinforcement learning or missing or biased medical data).

We identified a trend towards extensive use of methods based on decision trees, cluster analysis, and random forests. The popularity of these methods could be explained by the fact that they are generally associated with a greater potential for explainability of the results, which facilitates their translation into clinical practice. Other methods, such as support vector machine and recurrent neural networks, have also gained popularity in recent years, as they are known to show better performance for certain tasks (Paquette et al., 2021). Traditional statistical methods are also widely used. Regression methods remain especially popular for comparing the performance of AI methods, and correlation analysis may be useful for data preparation, such as excluding highly correlated variables to reduce the dimensions of the dataset. Although both types of methods (AI and statistical) produce useful results, no general framework exists for practical use. Future studies using AI models should therefore also include traditional statistical methods (e.g., Poisson or logistic regressions when using random forests or neural networks) in order to better define conditions of performance and to help create guidelines of AI methods applied to health care administrative data.

The majority of the articles covered medical diagnoses and treatment, which corresponds to the keywords used for the search in literature databases. Many studies were focused on hospital data and emergency departments that can be explained by better accumulation of data by large hospitals that are often affiliated to universities, and their better accessibility for AI research. These findings point to the need to improve data collection and accessibility for scientific research of outpatient data.

Interestingly, the word cloud revealed that different features describing socio-demographic characteristics were of different levels of interest in the reviewed studies. For example, “age” of patients was mentioned more frequently than “race”. Terms “gender” was found more frequently than “sex”, however, we think that in the context of health care administrative databases, those terms might be inter-replaceable, and most likely represent the value of sex. We decided not to replace them with one synonym to illustrate the weakness of currently available databases that do not allow distinguishing between these socio-demographic categories. Our results also revealed a wide range of features with an imbalance in the levels of interest (e.g., hypertension vs. pneumonia).

The most common health topics were related to chronic conditions including cardiovascular, renal, and respiratory diseases, cancer, and diabetes. The high number of research publications focused on these topics may be explained by their high prevalence in the population and heavy burden on the health care system, as well as the availability of surveillance data (Blais et al., 2014; Ke et al., 2022). These findings also correspond to the most frequent medications mentioned in the articles, such as metformin, heparin, and simvastatin. The commonly used term “insulin” could refer not only to pharmacotherapy, but also to clinical conditions such as insulin resistance or even to laboratory tests. However, our automatic analysis used to extract data, in its current form, does not allow distinguishing the contexts in which the term “insulin” was used in the articles.

### Strengths and limitations of the study

A librarian was involved in all stages of the review to define our search strategy and to strengthen the review process. The large number of abstracts included in the review helped to provide a global view on the state of the art of AI applied to health care administrative data that would focus on clinical problems and/or pharmacotherapy, as well as on the results of the treatment. However, manual data extraction from such a large number of articles was complicated and could have led to human errors. To avoid these errors and to facilitate data extraction at the last stage of the review we applied NLP-based method to automatize the procedure.

This search was limited to single-word terms and common acronyms of multi-word terms, and therefore some health and pharmacotherapy-related data might have been missed. Exclusion of studies based on databases with fewer than 1,000 observations could exclude studies using AI methods for a narrow or highly specific patient population. We suspected that the number of such studies excluded is small as AI methods are commonly used in large real-world databases.

## Conclusion

This scoping review revealed the potential of AI application to health-related studies. The most popular health areas for the application of AI include prediction of health outcomes and handling of large health datasets. Some areas of great importance for pharmacoepidemiology are, however, under-represented, such as health of specific socio-demographic groups and health effects of polypharmacy. With AI methods becoming increasingly popular, the extent to which they add value (in terms of efficiency, precision, or impact) is yet to be clarified.

## References

[B1] Azari A. Janeja V. Mohseni A. (2012). Predicting hospital length of stay (phlos): A multi-tiered data mining approach. IEEE 12th International Conference on Data Mining Workshops. 17–24. 10.1109/ICDMW.2012.69

[B2] Bagattini F. Karlsson I. Rebane J. Papapetrou P. (2019). A classification framework for exploiting sparse multi-variate temporal features with application to adverse drug event detection in medical records. BMC Med. Inf. Decis. Mak. 19, 7. 10.1186/s12911-018-0717-4 PMC6327495 30630486

[B3] Bates D. W. Saria S. Ohno-Machado L. Shah A. Escobar G. (2014). Big data in health care: Using analytics to identify and manage high-risk and high-cost patients. Health Aff. 33 (7), 1123–1131. 10.1377/hlthaff.2014.0041 25006137

[B4] Blais C. Jean S. Sirois C. Rochette L. Plante C. Larocque I. (2014). Quebec integrated chronic disease surveillance system (QICDSS), an innovative approach. Chronic Dis. Inj. Can. 34 (4), 226–235. 10.24095/hpcdp.34.4.06 25408182

[B5] Bueno M. L. P. Hommersom A. Lucas P. J. F. Lobo M. Rodrigues P. P. (2018). Modeling the dynamics of multiple disease occurrence by latent states. Scalable Uncertain. Manag. 11142, 93–107. 10.1007/978-3-030-00461-3_7

[B6] Chasioti D. Yao X. Zhang P. Lerner S. Quinney S. K. Ning X. (2019). Mining directional drug interaction effects on myopathy using the FAERS database. IEEE J. Biomed. Health Inf. 23 (5), 2156–2163. 10.1109/JBHI.2018.2874533 PMC6745690 30296244

[B7] Davazdahemami B. Delen D. (2019a). Examining the effect of prescription sequence on developing adverse drug reactions: The case of renal failure in diabetic patients. Int. J. Med. Inf. 125, 62–70. 10.1016/j.ijmedinf.2019.02.010 30914182

[B8] Davazdahemami B. Delen D. (2019b). The confounding role of common diabetes medications in developing acute renal failure: A data mining approach with emphasis on drug-drug interactions. Expert Syst. Appl. 123, 168–177. 10.1016/j.eswa.2019.01.006

[B9] Esteban C. Schmidt D. Krompass D. Tresp V. Balakrishnan P. Srivatsava J. (2015). Predicting sequences of clinical events by using a personalized temporal latent embedding model. Ieee. Int. Conf. Biomed. Health. Inf. 2130-139. 10.1109/ICHI.2015.23

[B10] Gubbi J. Buyya R. Marusic S. Palaniswami M. (2013). Internet of things (IoT): A vision, architectural elements, and future directions. Future Gener. Comput. Syst. 29 (7), 1645–1660. 10.1016/j.future.2013.01.010

[B11] Han X. Jiang F. Zhou H. Needleman J. Guo M. Chen Y. (2020). Hospitalization pattern, inpatient service utilization and quality of care in patients with alcohol use disorder: A sequence analysis of discharge medical records. Alcohol Alcohol 55 (2), 179–186. 10.1093/alcalc/agz081 31845973

[B12] Hung C. Y. Chen W. C. Lai P. T. Lin C. H. Lee C. C. (2017). Comparing deep neural network and other machine learning algorithms for stroke prediction in a large-scale population-based electronic medical claims database. Annu. Int. Conf. IEEE Eng. Med. Biol. Soc. 3110–3113. 10.1109/EMBC.2017.8037515 29060556

[B13] Jin H. Chen J. He H. Williams G. J. Kelman C. O' Keefe C. M. (2008). Mining unexpected temporal associations: Applications in detecting adverse drug reactions. IEEE Trans. Inf. Technol. Biomed. 124, 488–500. 10.1109/TITB.2007.900808 18632329

[B14] Ke C. Liang J. Liu M. Liu S. Wang C. (2022). Burden of chronic kidney disease and its risk-attributable burden in 137 low-and middle-income countries, 1990–2019: Results from the global burden of disease study 2019. BMC Nephrol. 23, 17. 10.1186/s12882-021-02597-3 34986789 PMC8727977

[B15] Khan A. Srinivasan U. Uddin S. (2019). Development and exploration of polymedication network from pharmaceutical and medicare benefits scheme data. ACSW 34, 1–6. 10.1145/3290688.3290738

[B16] Kone Pefoyo A. J. Rivard M. Laurier C. (2009). [Public health surveillance and role of administrative data]. Rev. Epidemiol. Sante Publique 57 (2), 99–111. 10.1016/j.respe.2008.11.003 19307073

[B17] Liu B. Li Y. Ghosh S. Sun Z. Ng K. Hu J. (2020). Complication risk profiling in diabetes care: A bayesian multi-task and feature relationship learning approach. IEEE Trans. Knowl. Data Eng. 32 (7), 1276–1289. 10.1109/TKDE.2019.2904060

[B18] Lopez-de-Andres A. Hernandez-Barrera V. Lopez R. Martin-Junco P. Jimenez-Trujillo I. Alvaro-Meca A. (2016). Predictors of in-hospital mortality following major lower extremity amputations in type 2 diabetic patients using artificial neural networks. BMC Med. Res. Methodol. 16 (1), 160. 10.1186/s12874-016-0265-5 27876006 PMC5120563

[B19] Mehta N. Pandit A. Shukla S. (2019). Transforming healthcare with big data analytics and artificial intelligence: A systematic mapping study. J. Biomed. Inf. 100, 103311. 10.1016/j.jbi.2019.103311 31629922

[B20] Melton L. J. Atkinson E. J. St Sauver J. L. Achenbach S. J. Therneau T. M. Rocca W. A. (2014). Predictors of excess mortality after fracture: A population-based cohort study. J. Bone Min. Res. 29 (7), 1681–1690. 10.1002/jbmr.2193 PMC4133107 24677169

[B21] Paquette F. X. Ghassemi A. Bukhtiyarova O. Cisse M. Gagnon N. Vecchia A. D. (2021). Machine learning support for decision making in kidney transplantation: Step-by-step development of a technological solution. JMIR Med. Inf. 10, e34554. 10.2196/34554 PMC9240927 35700006

[B22] Powell G. A. Verma A. Yu L. Stephens D. Buckeridge D. (2019). Modeling chronic obstructive pulmonary disease progression using continuous—time hidden Markov models. Stud. Health Technol. Inf. 264, 920–924. 10.3233/SHTI190358 31438058

[B23] Raghupathi W. Raghupathi V. (2014). Big data analytics in healthcare: Promise and potential. Health Inf. Sci. Syst. 2, 3. 10.1186/2047-2501-2-3 25825667 PMC4341817

[B24] Santana-Velásquez A. Duitama M. J. F. Arias-Londoño J. D. (2020). Classification of diagnosis-related groups using computational intelligence techniques. 2020 IEEE Colombian Conference on Applications of Computational Intelligence. 1–6. 10.1109/ColCACI50549.2020.9247889

[B25] Sessa M. Liang D. Khan A. R. Kulahci M. Andersen M. (2021). Artificial intelligence in pharmacoepidemiology: A systematic review. Part 2—comparison of the performance of artificial intelligence and traditional pharmacoepidemiological techniques. Front. Pharmacol. 11, 568659. 10.3389/fphar.2020.568659 33519433 PMC7841344

[B26] Shao Y. Ahmed A. Liappis A. P. Faselis C. Nelson S. J. Zeng-Treitler Q. (2021). Understanding demographic risk factors for adverse outcomes in COVID-19 patients: Explanation of a deep learning model. J. Healthc. Inf. Res. 5, 181–200. 10.1007/s41666-021-00093-9 PMC7914049 33681695

[B27] Timofte D. Stoian A. P. Razvan H. Diaconu C. Bulgaru-Iliescu D. Balan G. G. (2018). Postponed reconstructive surgery for entero-cutaneous fistulas. Surgery. 14, 13. 10.7438/1584-9341-14-3-1

[B28] Wang H. H. Wang Y. H. Liang C. W. Li Y. C. (2019a). Assessment of deep learning using nonimaging information and sequential medical records to develop a prediction model for nonmelanoma skin cancer. JAMA Dermatol. 155 (11), 1277–1283. 10.1001/jamadermatol.2019.2335 31483437 PMC6727683

[B29] Wang Y. H. Nguyen P. A. Islam M. M. Li Y. C. Yang H. C. (2019b). Development of deep learning algorithm for detection of colorectal cancer in EHR data. Stud. Health Technol. Inf. 264, 438–441. 10.3233/SHTI190259 31437961

[B30] Yang C. Delcher C. Shenkman E. Ranka S. (2016). Predicting 30-day all-cause readmissions from hospital inpatient discharge data. IEEE 18th Int. Conf. Heal., 1–6. 10.1109/HealthCom.2016.7749452

[B31] Yu k. Xie X. (2020). Predicting hospital readmission: A joint ensemble-learning model. IEEE J. Biomed. Health Inf. 24 (2), 447–456. 10.1109/JBHI.2019.2938995 31484143

[B32] Zhang J. Kowsari K. Harrison J. H. Lobo J. M. Barnes L. E. (2018). Patient2Vec: A personalized interpretable deep representation of the longitudinal electronic health record. IEEE Access 6, 65333–65346. 10.1109/access.2018.2875677

