# Supplementary material for: Major areas of interest of artificial intelligence research applied to health care administrative data: a scoping review
Source: Front Pharmacol. 2022 Jul 18;13:944516. doi: 10.3389/fphar.2022.944516 (PMC9340156; doi:10.3389/fphar.2022.944516)
Supplement: Supplementary file 1 [file DataSheet1.PDF]

Supplementary material – Database search strategy

Medline (Ovid)

Date of the search: 15-03-2021
Database limit: No database limit has been apply

Concepts	#	Search strategy	Results
Artificial intelligence (Controlled vocabulary)	1	Artificial Intelligence/ OR Algorithms/ OR "Neural Networks, Computer"/ OR exp "Machine Learning"/ OR Data Mining/ OR Cluster Analysis/	366793
Artificial intelligence (Free vocabulary)	2	((artificial OR computational) adj2 intelligence).ti,ab,kw OR Algorithm*.ti,ab,kw OR "auto-encoder".ti,ab,kw OR Autoencoder.ti,ab,kw OR ((Machine? OR Supervised OR Unsupervised OR Reinforcement OR Deep) adj2 Learning).ti,ab,kw OR Bayes*.ti,ab,kw OR "hidden Markov model".ti,ab,kw OR (Feature adj2 (learning OR selection)).ti,ab,kw OR ((Data OR Text) adj2 Mining).ti,ab,kw OR Partitioning.ti,ab,kw OR Graph*.ti,ab,kw OR "cluster analysis".ti,ab,kw OR "Neural Networks".ti,ab,kw	580203
Artificial intelligence (Combined)	3	1 OR 2	780979
Health data (Controlled vocabulary)	4	Electronic Health Records/ OR Medical Records/ OR Routinely Collected Health Data/ OR Health Records, Personal/ OR Health Surveys/ OR Patient Health Questionnaire/ OR big data/ OR Surveys and Questionnaires/	485860
Health data (Free vocabulary)	5	("Health administrative" adj2 data*).ti,ab,kw OR ((health OR healthcare) adj2 data).ti,ab,kw OR ((Medical OR Health OR patient) adj2 Record?).ti,ab,kw OR (Patient adj1 (representation OR characteristics)).ti,ab,kw OR (Health adj2 (survey? OR questionnaire?)).ti,ab,kw OR "big data".ti,ab,kw	281346
Health data (combined)	6	4 OR 5	739520
Outcomes of treatment (Controlled vocabulary)	7	exp Comorbidity/ OR Multiple Chronic Conditions/ OR Treatment Outcome/ OR exp "Drug-Related Side Effects and Adverse Reactions"/ OR Drug Interactions/ OR Pharmacovigilance/ OR Polypharmacy/ OR exp drug interaction/ OR Drug Therapy/ OR "Drug Therapy, Combination"/ OR "Potentially Inappropriate Medication List"/ OR Inappropriate Prescribing/ OR Decision Support Systems, Clinical/ OR Patient Care Management/ OR Critical Pathways/ OR Workflow/	1551262
Outcomes of treatment (Free vocabulary)	8	Multimorbid*.ti,ab,kw OR Comorbid*.ti,ab,kw OR ((concurrent OR multiple OR simultaneous) adj2 (chronic*) adj2 (diseases OR disorders OR conditions OR illnesses)).ti,ab,kw OR ((treat* OR clinical) adj2 (outcome? OR response)).ti,ab,kw OR "patient? outcome?".ti,ab,kw OR "Adverse drug reaction".ti,ab,kw OR "Drug Side Effect?".ti,ab,kw OR "Drug-drug interaction?".ti,ab,kw OR "Drug-disease interaction?".ti,ab,kw OR Pharmacovigilance.ti,ab,kw OR ((combination OR combined) adj2 ("drug treatment" OR pharmacotherapy OR "drug therapy")).ti,ab,kw OR "Drug Polytherap*".ti,ab,kw OR polypharmac*.ti,ab,kw OR ((concomitant* OR concurrent* OR multiple*) adj2 (medicine* OR medicat* OR prescrib* OR prescription* OR drug* OR pharmacoth*)).ti,ab,kw OR Pharmacotherap*.ti,ab,kw OR "Drug Therap*".ti,ab,kw OR ((Inappropriate OR over) adj2 (Prescribing OR medication?)).ti,ab,kw OR "STOPP START Criteria?".ti,ab,kw OR "Clinical decision support".ti,ab,kw OR "workflow management".ti,ab,kw OR "Healthcare pathway?".ti,ab,kw OR "Patient Journey".ti,ab,kw OR ((Clinical OR medical OR Healthcare OR model*) adj3 processes).ti,ab,kw	723085

Outcomes of treatment (combined)	9	7 OR 8	2045116
Review filter	10	Systematic Review/ OR Review/ OR Meta-Analysis/ OR meta-analysis as topic/ OR "Review Literature as Topic"/ OR "systematic review (topic)"/ OR ((literature* OR integrative OR map* OR narrative* OR "State-of-the-art" OR rapid? OR realist? OR systemati* OR umbrella*) adj3 review*).ti,ab,kw OR (scoping adj2 (stud* OR review)).ti,ab,kw OR (evidence adj2 map*).ti,ab,kw OR (("mixed stud*" OR "mixed method*" OR "meta-narrative*") adj3 (review* or synthes*)).ti,ab,kw OR ("Environmental scan*" OR "systematic map*" OR "evidence synthesis*" OR "meta-ethnograph*" OR "review of reviews").ti,ab,kw	3052692
Combination of concepts	11	(3 AND 6 AND 9) NOT 10	3448
Studies conducted between 2001 and 2021	12	limit 11 to yr="2001-2021"	3325

Embase (Embase.com)
Date of the search: 15-03-2021
Database limit: Embase results only

Concepts	#	Search strategy	Results
Artificial intelligence (Controlled vocabulary)	1	'artificial intelligence'/de OR 'artificial neural network'/de OR 'deep neural network'/exp OR 'machine learning'/exp OR 'autoencoder'/exp OR 'algorithm'/de OR 'cluster analysis'/de	548,284
Artificial intelligence (Free vocabulary)	2	((artificial OR computational) NEAR/2 intelligence):ti,ab,kw OR Algorithm*:ti,ab,kw OR "auto-encoder":ti,ab,kw OR Autoencoder:ti,ab,kw OR ((Machine$ OR Supervised OR Unsupervised OR Reinforcement OR Deep) NEAR/2 Learning):ti,ab,kw OR Bayes*:ti,ab,kw OR "hidden Markov model":ti,ab,kw OR (Feature NEAR/2 (learning OR selection)):ti,ab,kw OR ((Data OR Text) NEAR/2 Mining):ti,ab,kw OR Partitioning:ti,ab,kw OR Graph*:ti,ab,kw OR "cluster analysis":ti,ab,kw OR "Neural Networks":ti,ab,kw	723,007
Artificial intelligence (Combined)	3	#1 OR #2	947,714
Health data (Controlled vocabulary)	4	'health data'/de OR 'administrative health data'/de OR 'electronic health record'/de OR 'electronic medical record system'/de OR 'medical record'/exp OR 'health survey'/de OR 'questionnaire'/de OR 'patient health questionnaire'/de OR 'big data'/de	1,164,241
Health data (Free vocabulary)	5	("Health administrative" NEAR/2 data*):ti,ab,kw OR ((health OR healthcare) NEAR/2 data):ti,ab,kw OR ((Medical OR Health OR patient) NEAR/2 Record$):ti,ab,kw OR (Patient NEAR/1 (representation OR characteristics)):ti,ab,kw OR (Health NEAR/2 (survey$ OR questionnaire$)):ti,ab,kw OR "big data":ti,ab,kw	446,114
Health data (combined)	6	#4 OR #5	1,383,335
Outcomes of treatment (Controlled vocabulary)	7	'comorbidity'/de OR 'multiple chronic conditions'/de OR 'treatment outcome'/de OR 'clinical outcome'/de OR 'drug interaction'/exp OR 'adverse drug reaction'/de OR 'pharmacovigilance'/de OR 'polypharmacy'/de OR 'combination drug therapy'/de OR 'drug therapy'/de OR 'inappropriate prescribing'/exp OR 'clinical decision support system'/de OR 'health care management'/de OR 'managed care organization'/de OR 'patient care'/de OR 'workflow'/de OR 'clinical pathway'/de	2,885,187

Outcomes of treatment (Free vocabulary)	8	Multimorbid*:ti,ab,kw OR Comorbid*:ti,ab,kw OR ((concurrent OR multiple OR simultaneous) NEAR/2 (chronic*) NEAR/2 (diseases OR disorders OR conditions OR illnesses)):ti,ab,kw OR ((treat* OR clinical) NEAR/2 (outcome* OR response)):ti,ab,kw OR "patient outcome*":ti,ab,kw OR "Adverse drug reaction":ti,ab,kw OR "Drug Side Effect*":ti,ab,kw OR "Drug-drug interaction*":ti,ab,kw OR "Drug-disease interaction*":ti,ab,kw OR Pharmacovigilance:ti,ab,kw OR ((combination OR combined) NEAR/2 ("drug treatment" OR pharmacotherapy OR "drug therapy")):ti,ab,kw OR "Drug Polytherap*":ti,ab,kw OR polypharmac*:ti,ab,kw OR ((concomitant* OR concurrent* OR multiple*) NEAR/2 (medicine* OR medicat* OR prescrib* OR prescription* OR drug* OR pharmacoth*)):ti,ab,kw OR Pharmacotherap*:ti,ab,kw OR "Drug Therap*":ti,ab,kw OR ((Inappropriate OR over) NEAR/2 (Prescribing OR medication$)):ti,ab,kw OR "STOPP START Criteria$":ti,ab,kw OR "Clinical decision support":ti,ab,kw OR "workflow management":ti,ab,kw OR "Healthcare pathway$":ti,ab,kw OR "Patient Journey":ti,ab,kw OR ((Clinical OR medical OR Healthcare OR model*) NEAR/3 processes):ti,ab,kw	1,158,919
Outcomes of treatment (combined)	9	#7 OR #8	3,518,829
Review filter	10	'review'/exp OR 'meta analysis'/exp OR 'systematic review (topic)'/de OR 'meta analysis (topic)'/de OR ((literature* OR integrative OR map* OR narrative* OR "State-of-the-art" OR rapid$ OR realist$ OR systemati* OR umbrella*) NEAR/2 review*):ti,ab,kw OR (scoping NEAR/2 (stud* OR review)):ti,ab,kw OR (evidence NEAR/2 map*):ti,ab,kw OR (("mixed stud*" OR "mixed method*" OR "meta-narrative*") NEAR/3 (review* or synthes*)):ti,ab,kw OR ("Environmental scan*" OR "systematic map*" OR "evidence synthesis*" OR "meta-ethnograph*" OR "review of reviews"):ti,ab,kw	3,086,402
Combination of concepts	11	(#3 AND #6 AND #9) NOT #10	8,341
Embase results only	12	#11 AND [embase]/lim NOT ([embase]/lim AND [medline]/lim)	4,183
Studies conducted between 2001 and 2021	13	#12 AND [01-01-2001]/sd	4,174

CINAHL

Date of the search: 15-03-2021

Database limit: No database limit has been apply

Concepts	#	Search strategy	Results
Artificial intelligence (Controlled vocabulary)	1	MH "Artificial Intelligence" OR MH "Machine Learning" OR MH "Deep Learning" OR MH "Neural Networks (Computer)" OR MH "Data Mining" OR MH Algorithms OR MH "Cluster Analysis"	59,430
Artificial intelligence (Free vocabulary)	2	TI ((artificial OR computational) N2 intelligence) OR AB ((artificial OR computational) N2 intelligence) OR TI Algorithm* OR AB Algorithm* OR TI "auto-encoder" OR AB "auto-encoder" OR TI Autoencoder OR AB Autoencoder OR TI ((Machine? OR Supervised OR Unsupervised OR Reinforcement OR Deep) N2 Learning) OR AB ((Machine? OR Supervised OR Unsupervised OR Reinforcement OR Deep) N2 Learning) OR TI Bayes* OR AB Bayes* OR TI "hidden Markov model" OR AB "hidden Markov model" OR TI (Feature N2 (learning OR selection)) OR AB (Feature N2 (learning OR selection)) OR TI ((Data OR Text) N2 Mining) OR AB ((Data OR Text) N2	73,628

		Mining) OR TI Partitioning OR AB Partitioning OR TI Graph* OR AB Graph* OR TI "cluster analysis" OR AB "cluster analysis" OR TI "Neural Networks" OR AB "Neural Networks"	
Artificial intelligence (Combined)	3	S1 OR S2	107,957
Health data (Controlled vocabulary)	4	MH "Electronic Health Records" OR MH "Medical Records" OR MH "Medical Records, Personal" OR MH Surveys OR MH Questionnaires	561,285
Health data (Free vocabulary)	5	TI ("Health administrative" N2 data*) OR AB ("Health administrative" N2 data*) OR TI ((health OR healthcare) N2 data) OR AB ((health OR healthcare) N2 data) OR TI ((Medical OR Health OR patient) N2 Record?) OR AB ((Medical OR Health OR patient) N2 Record?) OR TI (Patient N1 (representation OR characteristics)) OR AB (Patient N1 (representation OR characteristics)) OR TI (Health N2 (survey? OR questionnaire?)) OR AB (Health N2 (survey? OR questionnaire?)) OR TI "big data" OR AB "big data"	140,092
Health data (combined)	6	S4 OR S5	654,450
Outcomes of treatment (Controlled vocabulary)	7	MH Comorbidity OR MH "Treatment Outcomes" OR MH "Drug Interactions+" OR MH Pharmacovigilance OR MH Polypharmacy OR MH "Drug Therapy" OR MH "Drug Therapy, Combination" OR MH "Inappropriate Prescribing" OR MH "Decision Support Systems, Clinical" OR MH "Patient Care" OR MH "Critical Path" OR MH "Patient Care" OR MH Workflow	534,178
Outcomes of treatment (Free vocabulary)	8	TI Multimorbid* OR AB Multimorbid* OR TI Comorbid* AB Comorbid* OR TI ((concurrent OR multiple OR simultaneous) N2 (chronic*) N2 (diseases OR disorders OR conditions OR illnesses)) OR AB ((concurrent OR multiple OR simultaneous) N2 (chronic*) N2 (diseases OR disorders OR conditions OR illnesses)) OR TI ((treat* OR clinical) N2 (outcome? OR response) OR AB ((treat* OR clinical) N2 (outcome? OR response) OR TI "patient? outcome?" OR AB "patient? outcome?" OR TI "Adverse drug reaction" OR AB "Adverse drug reaction" OR TI "Drug Side Effect?" OR AB "Drug Side Effect?" OR TI "Drug-drug interaction?" OR AB "Drug-drug interaction?" OR TI "Drug-disease interaction?" OR AB "Drug-disease interaction?" OR TI Pharmacovigilance OR AB Pharmacovigilance OR TI ((combination OR combined) N2 ("drug treatment" OR pharmacotherapy OR "drug therapy")) AB ((combination OR combined) N2 ("drug treatment" OR pharmacotherapy OR "drug therapy")) OR TI "Drug Polytherap*" OR AB "Drug Polytherap*" TI polypharmac* OR AB polypharmac* OR TI ((concomitant* OR concurrent* OR multiple*) N2 (medicine* OR medicat* OR prescrib* OR prescription* OR drug* OR pharmacoth*)) OR AB ((concomitant* OR concurrent* OR multiple*) N2 (medicine* OR medicat* OR prescrib* OR prescription* OR drug* OR pharmacoth*)) OR TI Pharmacotherap* OR AB Pharmacotherap* TI "Drug Therap*" OR AB "Drug Therap*" OR TI ((Inappropriate OR over) N2 (Prescribing OR medication?)) OR AB ((Inappropriate OR over) N2 (Prescribing OR medication?)) OR TI "STOPP START Criteria?" OR AB "STOPP START Criteria?" OR TI "Clinical decision support" OR AB "Clinical decision support" OR TI "workflow management" OR AB "workflow management" OR TI "Healthcare pathway?" OR AB "Healthcare pathway?" OR TI "Patient Journey" OR AB "Patient Journey" OR TI ((Clinical OR medical OR Healthcare OR model*) N3 processes) OR AB ((Clinical OR medical OR Healthcare OR model*) N3 processes)	163,247
Outcomes of treatment (combined)	9	S7 OR S8	641,466

Review filter	10	TI ((literature* OR integrative OR map* OR narrative* OR "State-of-the-art" OR rapid? OR realist? OR systemati* OR umbrella*) N2 review*)<br>OR AB ((literature* OR integrative OR map* OR narrative* OR "State-of-the-art" OR rapid? OR realist? OR systemati* OR umbrella*) N2 review*)<br>OR TI (scoping N2 (stud* OR review)) OR AB (scoping N2 (stud* OR review))<br>OR TI (evidence N2 map*) OR AB (evidence N2 map*)<br>OR TI (("mixed stud*" OR "mixed method*" OR "meta-narrative*") N3 (review* or synthes*))<br>OR TI ("Environmental scan*" OR "systematic map*" OR "evidence synthesis*" OR "meta-ethnograph*" OR "review of reviews")<br>OR AB ("Environmental scan*" OR "systematic map*" OR "evidence synthesis*" OR "meta-ethnograph*" OR "review of reviews")	201,645
Combination of concepts	11	(S3 AND S6 AND S9) NOT S10	2,153
Studies conducted between 2001 and 2021	12	S11 AND PY 2001-2021	2,108

Web of science

Date of the search: 15-03-2021

Database limit: Studies conducted between 2001 and 2021 limit has been apply

Concepts	#	Search strategy	Results
Artificial intelligence (Combined)	1	TS=((artificial OR computational) NEAR/2 intelligence) OR TS=(Algorithm*) OR TS=("auto-encoder") OR TS=(Autoencoder) OR TS=((Machine$ OR Supervised OR Unsupervised OR Reinforcement OR Deep) NEAR/2 Learning) OR TS=(Bayes*) OR TS=("hidden Markov model") OR TS=(Feature NEAR/2 (learning OR selection)) OR TS=((Data OR Text) NEAR/2 Mining) OR TS=(Partitioning) OR TS=(Graph*) OR TS=("cluster analysis") OR TS="Neural Networks"	3,314,268
Health data (combined)	2	TS=("Health administrative" NEAR/2 data*) OR TS=((health OR healthcare) NEAR/2 data) OR TS=((Medical OR Health OR patient) NEAR/2 Record$) OR TS=(Patient NEAR/1 (representation OR characteristics)) OR TS=(Health NEAR/2 (survey$ OR questionnaire$)) OR TS=("big data")	406,739
Outcomes of treatment (combined)	3	TS=Multimorbid* OR TS=Comorbid* OR TS=((concurrent OR multiple OR simultaneous) NEAR/2 (chronic*) NEAR/2 (diseases OR disorders OR conditions OR illnesses)) OR TS=((treat* OR clinical) NEAR/2 (outcome$ OR response)) OR TS="patient outcome$" OR TS="Adverse drug reaction" OR TS="Drug Side Effect$" OR TS="Drug-drug interaction$" OR TS="Drug-disease interaction$" OR TS=Pharmacovigilance OR TS=((combination OR combined) NEAR/2 ("drug treatment" OR pharmacotherapy OR "drug therapy")) OR TS=("Drug Polytherap*") OR TS=(polypharmac*) OR TS=((concomitant* OR concurrent* OR multiple*) NEAR/2 (medicine* OR medicat* OR prescrib* OR prescription* OR drug* OR pharmacoth*)) OR TS=(Pharmacotherap*) OR TS=("Drug Therap*") OR TS=((Inappropriate OR over) NEAR/2 (Prescribing OR medication$)) OR TS=("STOPP START Criteria$") OR TS=("Clinical decision support") OR TS=("workflow management") OR TS=("Healthcare pathway$") OR TS=("Patient Journey") OR TS=((Clinical OR medical OR Healthcare OR model*) NEAR/3 processes)	1,017,548
Review filter	4	TS=((literature* OR integrative OR map* OR narrative* OR "State-of-the-art" OR rapid$ OR realist$ OR systemati* OR umbrella*) NEAR/2 review*) OR TS=(scoping NEAR/2 (stud* OR review)) OR TS=(evidence NEAR/2 map*) OR TS=(("mixed stud*" OR "mixed method*" OR "meta-narrative*") NEAR/3	566,558

		(review* or synthes*)) OR TS=("Environmental scan*" OR "systematic map*" OR "evidence synthesis*" OR "meta-ethnograph*" OR "review of reviews")	
Combination of concepts	5	(#1 AND #2 AND #3) NOT #4	3,835

IEEE Xplore

Date of the search: 15-03-2021

Database limit: Journals, conferences prooceedings and studies conducted between 2001 and 2021 limits has been apply

Concepts	#	Search strategy	Results
Artificial intelligence (Combined)	1	"Artificial Intelligence" OR (Machine* NEAR/2 Learning) OR (Deep NEAR/2 Learning) OR (Data NEAR/2 Mining) OR (text NEAR/2 Mining) OR "auto-encoder"	385,834
Health data (combined)	2	(Health NEAR/2 data*) OR (healthcare NEAR/2 data) OR (Medical NEAR/2 Record*) OR (Health NEAR/2 Record*) OR (patient NEAR/2 Record*)	11,382
Combination of concepts	3	#1 AND #2	3,334
Journals, conferences prooceedings and studies conducted between 2001 and 2021 limits has been apply	4	n/a	3,171

ACM Digital Library (https://dl.acm.org/)

Date of the search: 15-03-2021

Database limit: no database limit has been apply

Concepts	#	Search strategy	Results
Combination of concepts	1	Abstract:(("Artificial Intelligence" OR "Machine* Learning" OR "Deep Learning" OR "Data Mining" OR "text Mining" OR "Neural Networks") OR title:("Artificial Intelligence" OR "Machine* Learning" OR "Deep Learning" OR "Data Mining" OR "text Mining" OR "Neural Networks")) AND Abstract:(("Health administrative data*" OR "health care data" OR "Healthcare data" OR "Health data" OR "Medical Record*" OR "Health Record*" OR "patient Record*" OR "Health survey*") OR "Health questionnaire*" OR "Patient representation" OR "Patient characteristics" OR title:("Health administrative data*" OR "health care data" OR "Healthcare data" OR "Health data" OR "Medical Record*" OR "Health Record*" OR "patient Record*" OR "Health survey*") OR "Health questionnaire*" OR "Patient representation" OR "Patient characteristics")	131

Compendex (Engineering village)

Date of the search: 15-03-2021

Database limit: Journals articles, journals in press and studies conducted between 2001 and 2021 limits has been apply

Concepts	#	Search strategy	Results

Artificial intelligence (Controlled vocabulary)	1	("Machine learning" WN CV OR "Neural networks" WN CV OR "Artificial intelligence" WN CV OR "Hidden Markov models" WN CV OR "Adaptive algorithms" WN CV OR "Learning algorithms" WN CV OR Algorithms WN CV OR "Recurrent neural networks" WN CV OR "Deep neural networks" WN CV OR "Deep learning" WN CV OR "Supervised learning" WN CV OR "Unsupervised learning" WN CV OR "Text mining" WN CV OR "Data mining" WN CV OR "Cluster analysis" WN CV OR "Graph neural networks" WN CV OR "Graph algorithms" WN CV)	1,320,024
Artificial intelligence (Free vocabulary)	2	("Artificial intelligence" WN KY OR "Computational Intelligence" WN KY OR Algorithm* WN KY OR "auto-encoder" WN KY OR Autoencoder WN KY OR (Machine* NEAR/2 Learning) WN KY OR (Supervised NEAR/2 Learning) WN KY OR (Unsupervised NEAR/2 Learning) WN KY OR (Reinforcement NEAR/2 Learning) WN KY OR (Deep NEAR/2 Learning) WN KY OR Bayes* WN KY OR "hidden Markov model" WN KY OR (Feature NEAR/2 learning) WN KY OR (Feature NEAR/2 selection) WN KY OR (Data NEAR/2 Mining) WN KY OR (Text NEAR/2 Mining) WN KY OR Partitioning WN KY OR Graph* WN KY OR "cluster analysis" WN KY OR "Neural Networks" WN KY OR Graph* WN KY OR Partitioning WN KY)	3,403,299
Artificial intelligence (Combined)	3	#1 OR #2	3,406,659
Health data (Controlled vocabulary)	4	"Medical information systems" WN CV OR "Big data" WN CV OR "Administrative data processing" WN CV	56,606
Health data (Free vocabulary)	5	(("Health administrative" NEAR/2 data) WN KY OR (health NEAR/2 data) WN KY OR (Medical NEAR/2 Record) WN KY OR (Health NEAR/2 Record*) WN KY OR (patient NEAR/2 Record*) WN KY OR (Patient NEAR/1 representation) WN KY OR (Patient NEAR/1 characteristics) WN KY OR (Health NEAR/2 survey) WN KY OR (Health NEAR/2 questionnaire) WN KY OR "big data" WN KY)	117,409
Health data (combined)	6	#4 OR #5	122,589
Outcomes of treatment (Controlled vocabulary)	7	("Patient treatment" WN CV OR "Drug therapy" WN CV OR "Drug interactions" WN CV OR "Health care" WN CV)	85,210
Outcomes of treatment (Free vocabulary)	8	Multimorbid* WN KY OR Comorbid* WN KY OR "treatment outcome*" WN KY OR "clinical outcome*" WN KY OR "clinical response" WN KY OR "patient outcome*" WN KY OR "Adverse drug reaction" WN KY OR "Drug Side Effect*" WN KY OR (Drug NEAR/2 interaction) WN KY OR Pharmacovigilance WN KY OR ("drug treatment" NEAR/2 combination) WN KY OR ("drug treatment" NEAR/2 combined) WN KY OR "drug therapy" WN KY OR pharmacotherapy WN KY OR "Drug Polytherap*" WN KY OR polypharmac* WN KY OR "Inappropriate Prescri*" WN KY OR "Inappropriate medication*" WN KY OR "over Prescri*" WN KY OR "over medication*" WN KY OR "STOPP START Criteria*" WN KY OR "Clinical decision support" WN KY OR "workflow management" WN KY OR "Healthcare pathway*" WN KY OR "Patient Journey" WN KY OR (Clinical NEAR/3 processes) WN KY OR (medical NEAR/3 processes) WN KY OR (Healthcare NEAR/3 processes) WN KY OR (model* NEAR/3 processes) WN KY	413,622
Outcomes of treatment (combined)	9	#7 OR #8	489,037

Review filter	10	(literature NEAR/2 review) WN KY OR (integrative NEAR/2 review) WN KY OR (mapping NEAR/2 review) WN KY OR (narrative NEAR/2 review) WN KY OR ("State-of-the-art" NEAR/2 review) WN KY OR (rapid NEAR/2 review) WN KY OR (realist NEAR/2 review) OR (systemati* NEAR/2 review) WN KY OR (umbrella NEAR/2 review) WN KY OR (scoping NEAR/2 review) WN KY OR (evidence NEAR/2 map) WN KY OR (evidence NEAR/2 mapping) WN KY OR ("mixed study" NEAR/3 review) WN KY OR ("mixed method" NEAR/3 review) WN KY OR ("meta-narrative*" NEAR/3 review) WN KY OR ("mixed study" NEAR/3 synthesis) WN KY OR ("mixed method" NEAR/3 synthesis) WN KY OR ("meta-narrative*" NEAR/3 synthesis) WN KY OR "Environmental scan*" WN KY OR "systematic map*" WN KY OR "evidence synthesis*" WN KY OR "meta-ethnograph*" WN KY OR "review of reviews" WN KY	67,923
Combination of concepts	11	(#3 AND #6 AND #9) NOT #10	4,667
Journals articles and journals in press limits	12	n/a	1,487
